# Clinical observation of Porcine Collagen Membrane + artificial Bovine Bone Granules Guided tissue regeneration combined with Autologous CGF in the treatment of severe periodontitis bone defect

**DOI:** 10.12669/pjms.39.3.6899

**Published:** 2023

**Authors:** Jue Cheng, Lin Cheng, Ling-Wei Kong, Wenfeng Qiu, Runtao Zhuang

**Affiliations:** 1 Jue Cheng, Department of Stomatology, Beijing Jiaotong University Hospital, Beijing 100000, Beijing, China; 2 Lin Cheng, Shanxi Bethune Hospital (Shanxi Academy of Medical Sciences), Taiyuan 030000, Shanxi, China; 3 Ling-Wei Kong, Department of Stomatology, Beijing Jiaotong University Hospital, Beijing 100000, Beijing, China; 4 Wenfeng Qiu, Department of Stomatology, Beijing Jiaotong University Hospital, Beijing 100000, Beijing, China; 5 Runtao Zhuang, Department of Stomatology, Beijing Jiaotong University Hospital, Beijing 100000, Beijing, China

**Keywords:** Porcine collagen membrane, Artificial bovine bone granules, Tissue regeneration, Autogenous concentrated growth factor, Periodontitis bone defect

## Abstract

**Objective:**

To investigate and analyze the clinical observation of porcine collagen membrane + artificial bovine bone granules guided tissue regeneration (GTR) combined with autologous concentration of growth factors (CGF) in the treatment of severe periodontitis bone defect.

**Methods:**

A total of 94 patients with severe periodontitis bone defects admitted to Shanxi Bethune Hospital from January 2019 to January 2022 were included. They were divided into two groups by simple randomization method. Patients in the control group were treated with porcine collagen membrane + artificial bovine bone granules GTR, while those in the observation group were treated with autologous CGF on the basis of the control group. Before and after treatment, periodontal clinical indicators [sulcus bleeding index (SBI), gingival retreat index (GR), probing depth (PD), clinical attachment loss (CAL), alveolar bone height (AH)] and bone resorption markers [Osteoprotegerin (OPG), bone gla protein (BGP), Type-1 collagen N-terminal peptide (NTX)] were compared between the two groups, and the incidence of postoperative complications in the two groups was recorded.

**Results:**

The total efficacy of observation group was significantly higher than that of control group (*p<*0.05). Three months after surgery, the observation group had lower levels of SBI, PD, CAL and NTX while higher levels of GR, AH, OPG and BGP than the control group (*p<*0.05). There was no significant difference in complication rate between the two groups (*p>*0.05).

**Conclusion:**

Porcine collagen membrane + artificial bovine bone granules GTR combined with autologous CGF boasts various benefits in the treatment of severe periodontitis bone defects, such as improvement of clinical outcomes, amelioration of periodontal tissue and inhibition of bone resorption.

## INTRODUCTION

Periodontitis, as one of the more common diseases in stomatology, is mainly caused by the inflammatory response of periodontal tissues due to various factors.^1^ Patients tend to ignore the disease due to its lack of specific symptoms, and often suffer from bone defects as the disease progresses and the inflammatory response spreads.^2^ In the case of severe periodontitis with bone defects, patients are exposed to the risk of tooth insufficiency and even tooth loosening, which seriously affects their quality of life.

Currently, regenerative treatment methods are often used clinically to promote the regeneration of periodontal tissue and restore the structure and function of periodontal tissue. However, relevant studies have reported^3^ that for patients with severe periodontitis bone defect, porcine collagen membrane + artificial bovine bone granules GTR alone is not so ideal in terms of curative effect, and varying degrees of graft material rejection will occur, affecting postoperative recovery. With the development and progress of medical technology and biomaterials in recent years, the autogenous concentration of growth factors (CGF) has been widely used in the restoration of dental implant bone defects and periodontal surgery, and achieved favorable outcomes.^4^,^5^

It is a novel blood extract that boasts higher concentrations of fibrin and promotes rapid healing of wound tissue. However, there are few clinical reports on porcine collagen membrane + artificial bovine bone granules GTR combined with autologous CGF in the treatment of severe periodontitis bone defects. We wished to investigate and analyze the clinical observation of porcine collagen membrane + artificial bovine bone granules guided tissue regeneration (GTR) combined with autologous concentration of growth factors (CGF) in the treatment of severe periodontitis bone defect.

## METHODS

This study included 94 patients with severe periodontitis bone defects admitted to Shanxi Bethune Hospital from January 2019 to January 2022. The study was approved by the Institutional Ethics Committee of Shanxi Bethune Hospital (Shanxi Academy of Medical Sciences) (No.:2021025; Date: May 10, 2021), and written informed consent was obtained from all participants.

### Inclusion criteria:

- Patients meeting the relevant diagnostic criteria of periodontitis;^6^
- Patients with probing depth (PD) ≥6 mm, teeth loosening of grade I-II, alveolar bone resorption length more than half of the root length after imaging examination, and degree of bone defect from the bottom of the bone pocket to the cementoenamel boundary of 9-12 mm.
- Patients who themselves and their families knew and gave informed consent to the study.

### Exclusion criteria:

- Patients with other dental pulp lesions;
- Patients with coagulation disorders and autoimmune diseases;
- Patients with blurred consciousness or mental illness;
- Patients with important organ diseases;
- Patients who cannot tolerate surgery.

All 94 patients were divided into two groups by simple randomization method: observation group (*n*=47) and control group (*n*=47). In the observation group, there were 21 males and 26 females, aged from 24 to 49 years, with an average of (36.73±3.61) years. The disease duration ranged from three to 10 months, with an average of (6.39±1.27) months. In the control group, there were 19 males and 28 females, aged from 24 to 49 years, with an average of (36.41±3.45) years. The course of the disease ranged from three to 10 months, with an average of (6.56±1.12) months. No statistically significant difference was observed in the general data comparison of the two groups (*p>*0.05).

### Surgical methods:

In both groups, a sulcus incision was made along the full thickness flap of affected teeth and bilateral adjacent teeth, and a thick flap was made on the membranous gingival part to preserve the gingival papilla as much as possible. The root of the affected tooth and the diseased area were completely exposed, the granulation was removed, the root surface was leveled, and the root surface was cleaned with normal saline after complete degeneration.

Patients in the control group were treated with porcine collagen membrane and artificial bovine bone granule guided tissue regeneration. First, bio-OSS cattle bone pellets (Bio-OSS, Geistlich, Switzerland) were filled into the bone defect and placed horizontally with the original bone. Then, the Bio-Gide collagen membrane (Geistlich, Switzerland) was cut to a suitable size according to the size of the bone defect, and the membrane should be about 2 mm beyond the bone defect. Finally, the diaphragm was covered over the bone defect and applied with appropriate pressure to the bone surface.

Patients in the observation group were treated with autologous CGF on the basis of the control group. *Preparation of autologous CGF*: 3 mL of venous blood was extracted from patients in the observation group before surgery and placed in a test tube without anticoagulant, and the test tube was placed in a centrifuge for 10 min (2200-3200 rpm). After the blood was divided into three layers (red blood cell and platelet layer, CGF gel fibrin clot and light yellow serum layer), the top layer of the light yellow serum layer was poured out, the CGF gel fibrin clot was removed with tweezers, and the red blood cell layer and plasma layer were cut off along the color junction. Finally, according to the needs of patients during surgery, CGF membranes were made or cut into pieces for use. *Operation:* Bio-OSS bovine bone particles and autologous CGF particles were mixed and filled into the bone defect area, which was horizontal with the original bone, then covered with autologous CGF membrane, and Bio-Gide collagen membrane was placed on the surface of the tooth root and alveolar bone, and the membrane should be about 2mm beyond the bone defect area.

All patients were operated on by the same doctor. They were routinely treated with antibiotics for 57 days after surgery, and gargled with compound chlorhexidine gargle. Stitches were removed one week later.

### Observation indicators: Efficacy evaluation^7^:

All patients were followed up three months after surgery and the clinical outcomes were evaluated. *Cured:* the patient’s pain, swelling and other symptoms disappeared completely, the periodontal condition returned to normal, no bone defect phenomenon; *Markedly effective:* pain, swelling and other symptoms of the patient disappeared, periodontal condition and bone defect were improved; Effective: patients with pain, swelling and other symptoms, periodontal status and bone defects were improved; Invalid: patients with pain, swelling and other symptoms, periodontal status and bone defects were not significantly improved or even aggravated. Total response rate= cure rate + markedly effective rate + effective rate.

### Periodontal clinical indicators:

One day before surgery and three months after surgery, periodontal clinical indicators such as sulcus bleeding index (SBI), gingival retreat index (GR), probing depth (PD), clinical attachment loss (CAL) and alveolar bone height (AH) were compared between the two groups.

### Bone resorption markers:

One day before surgery and three months after surgery, 3 mL of fasting venous blood was collected from the patient, and the samples were stored at -40°C. After the blood was completely coagulated, the serum was separated by centrifugation (8 cm centrifugation radius, 3000 r/min, 10 minutes). Osteoprotegerin (OPG), bone gla protein (BGP), Type-1 collagen N-terminal peptide (NTX) were determined by electrochemiluminescence immunoassay. Reagents and kits are matched with the instrument, and operation and testing are carried out strictly in accordance with the instructions.

### Complications:

postoperative complications, including periodontal redness, infection, periodontal pain and wound dehiscence, were recorded.

### Statistical processing:

All data in this study were processed and analyzed using SPSS version 22.0 statistical software. Periodontal clinical indicators, bone resorption markers and other measurement data that satisfy the normal distribution and have homogeneous variance are expressed as (±s). Paired *t-test* was used to compare the differences within, and the count data were expressed as n (%), and the chi-square test was used. P*<*0.05 indicates a statistically significant difference.

## RESULTS

The total efficacy of the observation group was 95.74%, which was significantly higher than 82.98% of the control group (*p<*0.05), as shown in Table-I. Before surgery, no statistically significant difference was observed in the levels of SBI, GR, PD, CAL and AH between the two groups (*p>*0.05). After surgery, the levels of SBI, PD and CAL in two groups were decreased, while GR and AH were increased. The levels of SBI, PD and CAL in the observation group were lower than those in the control group, while the levels of GR and AH were higher than those in the control group (*p<*0.05), as shown in Table-II.

**Table-I:** Comparison of the total clinical effective rate of the two groups (n, %).

Group	Number of cases	Cured	Markedly effective	Effective	Invalid	Total effective rate
Observation group	47	22	14	9	2	95.74
Control group	47	18	11	10	8	82.98
*x* ^2^			-	-	-	4.029
p			-	-	-	0.045

**Table-II:** Comparison of periodontal clinical indicators between the two groups (*χ̅*±*S*, *n*=47)

Indicators	Time	Observation group	Control group	t	p
SBI	Before surgery	1.33±0.31	1.41±0.40	1.084	0.281
	After surgery	0.89±0.24^*^	1.12±0.26^*^	4.456	<0.001
GR (mm)	Before surgery	1.19±0.34	1.28±0.35	1.264	0.209
	After surgery	2.27±0.63^*^	2.01±0.53^*^	2.165	0.033
PD (mm)	Before surgery	6.38±1.20	6.41±1.22	0.120	0.905
	After surgery	3.24±0.92^*^	3.69±0.96^*^	2.272	0.025
CAL (mm)	Before surgery	7.49±1.34	7.46±1.17	0.116	0.908
	After surgery	4.11±1.06^*^	4.87±1.25^*^	3.179	0.002
AH (mm)	Before surgery	7.22±1.09	7.33±1.15	0.476	0.635
	After surgery	10.76±2.51^*^	8.94±2.11*	3.805	<0.001

***Note:***

* p<0.05 compared with that before surgery.

Before surgery, no statistical significance was observed in the comparison of OPG, BGP and NTX levels between the two groups (*p>*0.05). After surgery, the levels of OPG and BGP in both groups increased, while NTX decreased. OPG and BGP in the observation group were higher than those in the control group, while NTX was lower than those in the control group (*p<*0.05), as shown in Table-III. The incidence of complications was 12.77% in the observation group and 17.02% in the control group, with no statistically significant difference (*p>*0.05), as shown in Table-IV.

**Table-III:** Comparison of bone resorption markers between the two groups (*χ̅*±*S*, *n*=47)

Group	OPG (ng/L)		BGP (ug/L)		NTX (nmol/L)	

	Before surgery	After surgery	Before surgery	After surgery	Before surgery	After surgery
Observation group	1.32±0.35	2.58±0.56*	3.85±0.26	6.68±1.39*	26.38±3.54	14.25±2.17*
Control group	1.39±0.31	2.17±0.52*	3.91±0.37	5.43±1.37*	26.56±3.16	16.49±2.20*
t	0.103	3.678	0.910	4.391	0.262	4.970
p	0.918	<0.001	0.365	<0.001	0.794	<0.001

***Note:***

* p<0.05 compared with that before surgery.

**Table-IV:** Comparison of postoperative complications between the two groups (n, %).

Group	Number of cases	Periodontal redness	Infection	Periodontal pain	Wound dehiscence	Complication rate
Observation group	47	1 (2.13)	2 (4.26)	2 (4.26)	1 (2.13)	6 (12.77)
Control group	47	2 (4.26)	3 (6.38)	1 (2.13)	2 (4.26)	8 (17.02)
*x* ^2^			-	-	-	0.336
p			-	-	-	0.562

Moreover, this surgical method has better coagulation and anti-inflammatory effects, boasting various benefits such as sealing wounds, reducing the risk of infection, further improving the clinical efficacy of patients, and promoting recovery.

## DISCUSSION

The observation group had lower levels of SBI, PD and CAL while higher levels of GR and AH than the control group, suggesting that porcine collagen membrane + artificial bovine bone granules guided tissue regeneration combined with autologous CGF is conducive to promoting the reconstruction of periodontal tissues in patients with severe periodontitis bone defects. The mesh-like fibrin structure of autologous CGF is adhesive, thin and soft, which easily allows growth factors and osteoblasts to migrate, proliferate and differentiate at the surface of the bone defect.^8^ Furthermore, the growth factors it released are synergistic with the restorative effect provided by the porcine collagen membrane + artificial bovine bone granules, which enhances tissue repair and angiogenesis, enabling the periodontal tissue rebuilt. OPG has the function of inhibiting the differentiation and maturation of osteoclasts; BGP level reflects the activity of osteoblasts; and NTX is a specific indicator of bone resorption in bone transformation.^9^ In this study, the levels of OPG and BGP in the observation group were significantly higher than those in the control group while the level of NTX was significantly lower than those in the control group, suggesting that porcine collagen membrane + artificial bovine bone granules guided tissue regeneration combined with autologous CGF could reduce bone resorption in the treatment of severe periodontitis bone defects. To explain the reason, autologous CGF with strong regenerative ability may significantly shorten the time of promoting osteogenesis in the operated area, improve the quality of osteogenesis, and promote osteogenesis and tissue healing; In addition, it may also induce hard tissue formation, enhance osteocyte activity, and inhibit osteoclast activity.^10.11^ Autologous CGF, combined with porcine collagen membrane + artificial bovine bone granules guide tissue regeneration, can efficiently induce bone regeneration, so that bone cells and blood vessels can be fully combined and grown. In this way, bone development can be further promoted, and bone resorption of periodontal tissue can be inhibited. The conclusion of this study provides a clinical reference for exploring the clinical treatment of bone defects in severe periodontitis.

With the change of lifestyle in recent years, periodontitis has witnessed a gradual upward trend in its prevalence. As periodontitis develops, it will be accompanied by alveolar bone destruction and resorption and loss of periodontal attachment, which will further lead to tooth loosening and even loss.^12^ Severe periodontitis bone defect is a formidable challenge for periodontal bone repair. Traditionally, periodontal replantation has been used to facilitate the rapid healing of bone defects and the formation of new periodontal attachments. However, postoperative complications tend to occur, and osteogenesis is time-consuming.^13^ For this reason, more appropriate surgical methods have a positive effect on improving the clinical efficacy of patients with severe periodontitis and bone defects.

Porcine collagen membrane + artificial bovine bone granules guided tissue regeneration, as a natural collagen barrier membrane, is mainly composed of 10% highly purified porcine collagen and natural bovine bone material, boasting features such as convenient operation, increased tissue stability, and promoted bone regeneration.^14^,^15^ However, when performing this procedure, patients are subject to their periodontal bone regeneration process being affected by the effect of the graft material. CGF is not only a plasma extract, but also a repair biomaterial that contains concentrated growth factors and fibrin, with the unique property of improving and enhancing tissue regeneration.^16^ In this study, the clinical efficacy of the observation group was significantly higher than that of the control group, indicating a high efficacy of porcine collagen membrane + artificial bovine bone granules guided tissue regeneration combined with autologous CGF in the treatment of severe periodontitis bone defects, which is conducive to the rapid recovery of patients after surgery. This may be due to the fact that autologous CGF is derived from the patient’s own venous blood. After being separated and prepared by a special centrifugation method, autologous CGF is injected in combination with porcine collagen membrane + artificial bovine bone granules into the patient’s periodontitis bone defect, which facilitates the repair of bone defects, induces growth, accelerates wound healing and improves the quality of healing.^17^,^18^

### Limitations of this study:

It includes a small number of samples were included, and the results were subject to selection bias. In future more patients should be included for more in-depth research in clinical practice.

## CONCLUSION

Porcine collagen membrane + artificial bovine bone granules GTR combined with autologous CGF boasts various clinical effects in the treatment of severe periodontitis bone defects, such as promoting periodontal tissue reconstruction and reducing bone resorption.

### Authors’ Contributions:

**JC** and **LC** designed this study, prepared this manuscript, are responsible and accountable for the accuracy and integrity of the work.

**LWK** and **WQ** collected and analyzed clinical data.

**RZ** Data analysis**,** significantly revised this manuscript.
